# Chrysin Inhibits Tumor Promoter-Induced MMP-9 Expression by Blocking AP-1 via Suppression of ERK and JNK Pathways in Gastric Cancer Cells

**DOI:** 10.1371/journal.pone.0124007

**Published:** 2015-04-15

**Authors:** Yong Xia, Sen Lian, Pham Ngoc Khoi, Hyun Joong Yoon, Young Eun Joo, Kee Oh Chay, Kyung Keun Kim, Young Do Jung

**Affiliations:** Research Institute of Medical Sciences, Chonnam National University Medical School, Gwangju, Republic of Korea; University of Nebraska Medical Center, UNITED STATES

## Abstract

Cell invasion is a crucial mechanism of cancer metastasis and malignancy. Matrix metalloproteinase-9 (MMP-9) is an important proteolytic enzyme involved in the cancer cell invasion process. High expression levels of MMP-9 in gastric cancer positively correlate with tumor aggressiveness and have a significant negative correlation with patients’ survival times. Recently, mechanisms suppressing MMP-9 by phytochemicals have become increasingly investigated. Chrysin, a naturally occurring chemical in plants, has been reported to suppress tumor metastasis. However, the effects of chrysin on MMP-9 expression in gastric cancer have not been well studied. In the present study, we tested the effects of chrysin on MMP-9 expression in gastric cancer cells, and determined its underlying mechanism. We examined the effects of chrysin on MMP-9 expression and activity via RT-PCR, zymography, promoter study, and western blotting in human gastric cancer AGS cells. Chrysin inhibited phorbol-12-myristate 13-acetate (PMA)-induced MMP-9 expression in a dose-dependent manner. Using AP-1 decoy oligodeoxynucleotides, we confirmed that AP-1 was the crucial transcriptional factor for MMP-9 expression. Chrysin blocked AP-1 via suppression of the phosphorylation of c-Jun and c-Fos through blocking the JNK1/2 and ERK1/2 pathways. Furthermore, AGS cells pretreated with PMA showed markedly enhanced invasiveness, which was partially abrogated by chrysin and MMP-9 antibody. Our results suggest that chrysin may exert at least part of its anticancer effect by controlling MMP-9 expression through suppression of AP-1 activity via a block of the JNK1/2 and ERK1/2 signaling pathways in gastric cancer AGS cells.

## Introduction

Gastric cancer currently ranks second in global cancer mortality, with an estimated 990,000 new cases and 738,000 cancer deaths resulting worldwide annually, although the incidence of stomach carcinoma has decreased in the past few decades [1,2]. Distant organ or tissue metastasis is a sign of poor prognosis in patients with gastric cancer. Metastasis is the most fatal characteristic of malignant tumors, accounting for more than 90% of tumor-related mortalities [2]. It has been shown that chemotherapy and radiation therapy cannot significantly prolong and improve the quality of life of patients in those instances [3,4]. Tumor cells metastasis is a very complex process comprising of proliferation, migration, invasion, and the subsequent adhesion and angiogenesis in other organs or tissues [3].

Since invasion is one of the fundamental properties of malignant cancer cells, controlling invasion, is an important therapeutic target. Cell-extracellular matrix (ECM) interactions, disconnection of intercellular adhesion, degradation of the ECM, and the invasion of lymph and blood vessels are important steps in cancer invasion and metastasis [5]. Tumor invasion requires an increased expression of matrix metalloproteinases (MMPs) [6]. MMPs, a family of zinc-dependent endopeptidases which induce cancer cell invasion and spread, play crucial roles in metastasis through the degradation of the ECM and the basal membrane [7]. Matrix metalloproteinase-9 (MMP-9), known as 92 kDa type-IV collagenase or gelatinase B, is one of the most important MMPs, and is encoded by the MMP-9 gene in humans [8]. It has been reported that overexpression of MMPs can increase tumor cell detachment and metastasis, which are associated with malignancy and poor clinical outcomes in various cancers including gastric cancer [9,10].

Due to the function of MMP-9 in the course of malignancy, the suppression of MMP-9 levels is an important strategy for controlling cancer. In the recent years, more and more attention has been focused on finding an MMP-9 inhibitor, especially from naturally occurring materials. Kim et al. discovered that silibinin inhibits PMA-induced MMP-9 expression through suppression of ERK phosphorylation in MCF-7 human breast cancer cells [11]. More recently, Khoi et al. reported that (-)-Epigallocatechin-3-gallate blocks nicotine-induced MMP-9 expression and invasiveness through the suppression of NF-κB and AP-1 in endothelial ECV304 cells [12]. Chrysin, 5,7-dihydroxyflavone, a type of naturally occurring flavonoid, has been known to inhibit angiogenesis and metastasis [13,14]. Lin et al. demonstrated that chrysin suppresses IL-6-induced angiogenesis through down-regulation of the soluble IL-6 receptor/gp130/JAK1/STAT3/VEGF signaling pathway [13]. Recently it is reported that chrysin could enhance the caspase-dependent apoptosis regulated by TRAIL in HCT 116 cell line and CNE1 cells [15]. Yang et al. discovered that chrysin suppressed cell invasion in a dose-dependent manner in TNBC cells. Moreover, chrysin decreases metastasis-related molecules vimentin, snail and slug, and blocks the Akt signaling pathway [16]. However, the inhibitory effects of chrysin on MMP-9, as well as the mechanism, have not been well studied, especially in gastric cancer cells. In the present study, we investigated chrysin’s effects on PMA-induced MMP-9 expression in gastric cancer, and revealed its underlying mechanism.

## Materials and Methods

### Cell culture and culture conditions

The AGS human gastric cancer cell line was obtained from the American Type Culture Collection (Manassas, VA, USA). The cells were cultured in RPMI-1640 supplemented with 10% fetal bovine serum (FBS) and 0.6% penicillin-streptomycin at 37°C in an atmosphere containing 5% CO_2_. To determine the effects of chrysin on PMA induced MMP-9 expression, the cells were pretreated with different concentrations of chrysin and then treated with PMA. The level of MMP-9 messenger RNA (mRNA) was determined by reverse transcription-polymerase chain reaction (RT-PCR) analysis. The role of the specific signaling pathways in the PMA-induced MMP-9 expression were examined by pretreating the AGS cells with the ERK 1/2 inhibitor PD98059 (New England Biolabs, Beverly, MA), the JNK inhibitor SP600125 (Calbiochem, La Jolla, CA), the p38 MAPK inhibitor SB203580 (Calbiochem, La Jolla, CA), and chrysin (Sigma Aldrich, USA) for 1 hour before stimulation with PMA.

### Cell viability

Cells (5×10^3^) were incubated in a 96-well plate in RPMI with 10% FBS containing 0–100 μM chrysin for 24 hours, and cell respiration was determined by an established 3-[4,5-dimethylthiazol-2-yl]-2,5-diphenyltetrazolium bromide (MTT; Sigma-Aldrich) assay. After the incubation, 10 μl of 5 mg/ml MTT was added to each well of the 96-well plates and incubated at 37°C for 2 hours. The formazan granules obtained were dissolved in 100% dimethyl sulfoxide, and the absorbance at 570 nm was detected with a 96-well ELISA reader (Biotek Inc., Winooski, VT, USA).

### Gelatin zymography

Cells pretreated with or without chrysin for 1 hour were treated with 100 nM PMA for 20 hours. After incubation, we collected the media supernatant and performed gelatin zymography to check the MMP-9 activity. Subsequently, the media was mixed with an equal volume of 2P-9 activityrazolium b [62.5 mM Tris-HCl (pH6.8), 25% glycerol, 4% SDS, and 0.01% bromophenol blue] and loaded into a 7.5% acrylamide:bisacrylamide (29:1; Bio-Rad, USA) gel containing 625 μg/ml gelatin (Sigma). After electrophoresis, the gel was washed with 2.5% Triton X-100 three times (20 minutes per time), then incubated for 24 hours at 37°C, in the incubation buffer [50 mM Tris (pH 7.5), 100 mM NaCl, 5 mM CaCl_2_, and 1 μM ZnCl_2_]. Gels were stained with Coomassie Brilliant Blue R250 (Bio-Rad, USA) for 3 hours, and then rinsed. Proteolytic activity was reflected as clear bands against the blue background of the stained gelatin. Loading control for gelatin zymography: Coomassie Brilliant Blue R-250 stain for 10% sodium dodecyl sulfate polyacrylamide gel without gelatin, to show equal amount of supernatant was loaded onto each lane.

### Reverse transcription-PCR

Total RNA was extracted from the AGS cells using TRIzol reagent (Invitrogen). One μg of total RNA was used for first-strand complementary DNA synthesis using random primers and M-MLV transcriptase (Promega). The complementary DNA was subjected to PCR amplification with primer sets for GAPDH and MMP-9 using a PCR master mix solution (iNtRON, Korea). The specific primers sequences were as follows: GAPDH sense, 5’-TTG TTG CCA TCA ATG ACC CC-3’ and GAPDH antisense, 5’-TGA CAA AGT GGT CGT TGA GG-3’ (836 bp); and MMP-9 sense, 5’- AAG TGG CAC CAC CAC AAC AT -3’ and MMP-9 antisense, 5’-TTT CCC ATC AGC ATT GCC GT-3’ (497 bp); c-Jun sense, 5’-GGA AAC GAC CTT CTA TGA CGA TGC CCTCAA-3’, and c-Jun antisense, 5’- GAA CCC CTC CTG CTC ATC TGT CAC GTT CTT-3’ (287bp); c-Fos sense, 5’-CAG TCA GAT CAA GGG AAG CCA CAG ACA TCT-3’ and c-Fos antisense, 5’-GAA TAA GAT GGC TGC AGC CAA ATG CCG CA-3’(246bp). The PCR conditions were as follows: denaturation at 94°C for 30 seconds, annealing at 52°C for 20 seconds and extension at 72°C for 30 seconds.

### Measurement of MMP-9 promoter activity

The transcriptional regulation of MMP-9 was examined by the transient transfection of an MMP-9 promoter–luciferase reporter construct (pGL4-MMP-9). The plasmid pGL4-MMP-9 promoter (spanning nucleotides from -925 to +13) was kindly provided by Dr. Young-Han Lee (Konkuk University, Korea). AGS cells were seeded and grown until they reached 70% confluence. Then, the pGL4-MMP-9 promoter plasmids were transfected into the cells using FuGENE 6 (Promega, USA) according to the manufacturer’s protocol. PRL-TK was transfected as an internal control. Cells were incubated in the transfection medium for 12 hours and then treated with PMA for 4 hours. The effects of chrysin on MMP-9 promoter activity were determined by pretreating cells with chrysin for 1 hour prior to addition of PMA. The co-transfection studies were performed in the presence or absence of the expression vector encoding the dominant negative mutant MEK-1 (pMCL-K97M), dominant negative mutant JNK (pMCL-TAM67), or dominant negative mutant p38 MAPK (pMCL-mP38) which were kindly provided by Dr. N.G. Ahn (University of Colorado-Boulder, CO), by Dr. M.J. Birrer (NCI, Rockville, MD), and by Dr. J. Han (Scripps Research Institute, CA), respectively. The cells were harvested with a cell culture lysis reagent (Promega, USA), and the luciferase activities were determined using a luminometer (Centro XS lb960 microplate luminometer, Berthold Technologies, USA) according to the manufacturer’s protocol.

### Transient transfection of AP-1 reporter

The AP-1 luciferase reporter plasmid was purchased from Clontech (Palo Alto, CA, USA). When AGS cells reached 60–70% confluence, they were washed with Opti-MEM medium and transfected with a pGL-3 vector containing the AP-1 reporter using FuGENE 6 (Promega) according to the manufacturer’s protocol. Reporter-transfected cells were pretreated with chrysin for 1 hour and then treated with 100 nM PMA for 4 hours and the luciferase activities were measured using a luminometer.

### Transfection of AP-1 decoy oligodeoxynucleotides

Based on the AP-1 binding site ATGAGTCAT, we designed the AP-1 decoy phosphorothioated double-stranded oligo deoxynucleotide (ODNs) as follows, 5’-CGsT CTT CTG AGT CAT GAA TTC ATG ACT CAG AAG ACsG-3’, and 3’-GsCA GAA GAC TCA GTA CTT AAG TAC TGA GTC TTC TsGC-5’. When the AGS cells grew to 60–70% confluence, AP-1 ODNs and pGL4-MMP-9 promoter plasmids were co-transfected into cells using Lipofectamine 2000 (Invitrogen, USA) according to the manufacturer’s protocol. After incubating for 20 hours, the transfected cells were treated with PMA for 4 hours and the luciferase activities were measured using a luminometer.

### Western blot analysis

AGS cells treated with PMA were washed in phosphate buffered saline (PBS), detached using Trypsin-EDTA buffer, and stored at -70°C until needed. The cellular protein was extracted with a RIPA buffer [1% NP-40, 0.5% sodium deoxycholate, 0.1% sodium dodecyl sulfate (SDS)] and protease inhibitors (aprotinin, leupeptin, phenylmethanesulfonylfluoride (PMSF), benzamidine, trypsin inhibitor, sodium orthovanadate). Fifty micrograms of the proteins was then separated by 10% SDS-polyacrylamide gel electrophoresis and transferred to Hybond-P membranes (Amersham Pharmacia Biotech). The membranes were blocked in a TBST solution containing 5% skim milk, incubated with a primary antibody in a blocking solution overnight at 4°C, and washed three times with 0.1% Tween-20 in Tris buffered saline (TBST) at 10 minutes intervals. Horseradish peroxidase-conjugated secondary antibody (Amersham, Arlington Heights, IL, USA) was used to detect the immunoreactive proteins by chemiluminescence. The following antibodies were used: anti-phospho-p44/42 MAPK (ERK-1/2) (Cell Signaling Technology, Danvers, MA, USA), anti-phospho-JNK/SAPK (Cell Signaling Technology), anti-MMP-9 (Cell Signaling Technology), anti-phospho-c-Fos (Cell Signaling Technology), and anti-phospho-c-Jun (Cell Signaling Technology). The total protein levels were assayed by washing the blotted membrane with a stripping solution composed of 100 mM 2-mercaptoethanol, 2% SDS, and 62.5 mM Tris–HCl (pH 6.7) for 30 minutes at 50°C, and the membrane was then re-probed with either the anti-β-actin (Cell Signaling Technology), anti-total ERK1/2 (Cell Signaling Technology), anti-total JNK/SAPK (Cell Signaling Technology), anti-c-Fos (Santa Cruz Biotechnology, Inc., CA, USA) or anti-c-Jun (Santa Cruz).

### Matrigel invasion assay

The cell invasion assay was carried out using the 10-well chemotasis chamber (Neuro Probe, Gaithersburg, Maryland, USA) with 8 μM pore membrane (Neuro Probe) with 10% FBS containing RPMI as the chemoattractant in the lower chamber. AGS cells (10^5^ in 280 μl) were added to upper chamber with PMA in the presence of chrysin, MMP-9 antibody or nonspecific IgG, and incubated to invade the Matrigel for 24 hours. In order to determine the effect of chrysin and signaling inhibitors on PMA-induced cell invasion, AGS cells were pre-incubated with chrysin, curcumin, PD98059, or SP600125 for 1 hour, and incubated with PMA for the 24 hour invasion period. The non-invading cells on the upper surface of each membrane were removed from the chamber, and the invading cells on the lower surface of each membrane were stained with the Quick-Diff stain kit (Becton-Dickinson, Franklin Lakes, NJ, USA). After two water washes, the chambers were allowed to air-dry. The number of invading cells was counted using a phase-contrast microscope.

### Statistics

Data are shown as mean ± SD, and represent the mean of at least three separate experiments performed in triplicate. Differences between every two data sets were determined by t-tests. Differences described as significant in the text correspond to *P*<0.05.

## Results

### Inhibitory effect of chrysin on PMA stimulated MMP-9 expression

**Fig 1:**
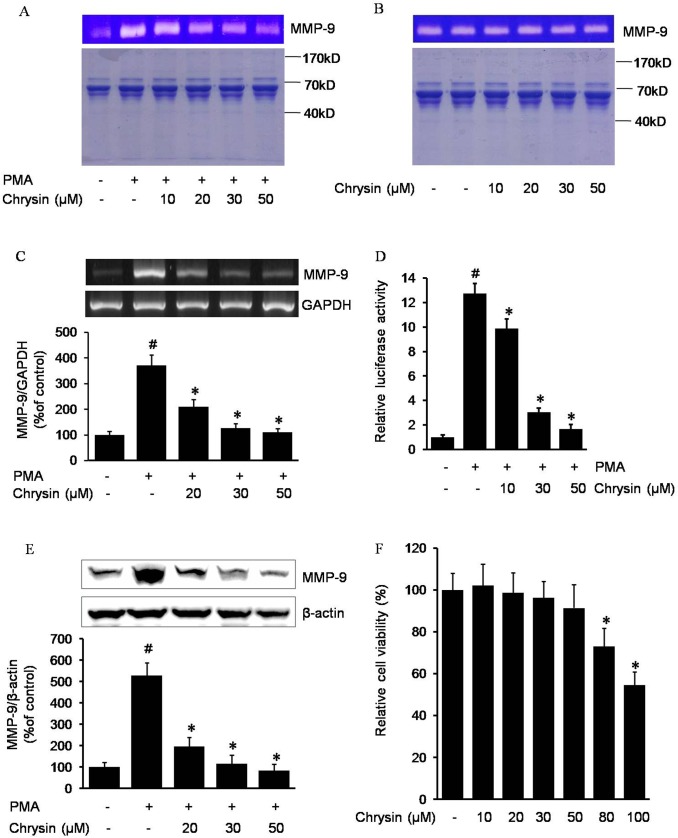
Chrysin inhibits PMA-induced MMP-9 in AGS cells. A total of 2×10^6^ cells pretreated with the indicated concentrations of chrysin for 1 h were treated with 100 nM PMA for 20 h. After incubation, cell culture media supernatant was collected and gelatin zymography was performed to analyze the MMP-9 activity (A). The cell culture media containing AGS cells secreted MMP-9 were incubated with different concentrations of chrysin in 37°C for 3 h, then gelatin zymography was performed to analyze the final MMP-9 activities (the first lane, loading a control, contained the cell culture media supernatant without incubation with chrysin) (B). Cells pretreated with the indicated concentrations of chrysin for 1 h were treated with 100 nM PMA for 4 h. After incubation, RT-PCR was performed to analyze the MMP-9 mRNA. # *P*<0.05 versus control; * *P*<0.05 versus only PMA (C). Cells were transiently transfected with a pGL4-MMP-9 promoter reporter construct. The transfected cells were pretreated with the indicated concentrations of chrysin for 1 h, then were incubated with 100 nM for 4 h, at which point luciferase activity was determined using a luminometer. # *P*<0.05 versus control; * *P*<0.05 versus only PMA (D). Cells pretreated with the indicated concentrations of chrysin for 1 h were treated with 100 nM PMA for 8 h. After incubation, western bolt was performed to analyze the MMP-9 protein level. # *P*<0.05 versus control; * *P*<0.05 versus only PMA (E). The cells were co-incubated with 0–80 μM chrysin for 24 h, and then their viabilities were tested by the MTT method. * *P*<0.05 versus control (without chrysin) (F). The data represents the mean ± SD from triplicate measurements.

To investigate the suppressive effect of chrysin against the upregulation of MMP-9, AGS cells pretreated with chrysin were incubated with PMA, and gelatin zymography and RT-PCR analysis were performed. As shown in Fig 1A, PMA-stimulated MMP-9 was inhibited by chrysin in a dose-dependent manner as illustrated via the gelatin zymography assay. Prior to our experiment, whether chrysin directly inhibited the activity of MMP-9 or inhibited the expression of MMP-9 was still unknown. To answer this question, we co-incubated the AGS-secreted MMP-9 with chrysin for 3 hours, and gelatin zymography was performed to check the final level of MMP-9 activity. As Fig 1B shows, chrysin could not affect secreted MMP-9 activities. So, we hypothesized that the inhibition of chrysin against MMP-9 may be via suppression of MMP-9 expression. To verify this hypothesis, reverse transcriptional PCR and MMP-9 promoter assays were performed to check the MMP-9 transcription levels. As shown in Fig 1C, after being treated with chrysin, the PMA-induced MMP-9 mRNA decreased in a dose-dependent manner. Furthermore, MMP-9 promoter luciferase activity was inhibited by chrysin in a dose-dependent manner (Fig 1D). And western blotting also showed the inhibitory effect of chrysin on MMP-9 expression (Fig 1E). The concentrations of chrysin used in the present study did not affect cell viability (Fig 1F). These results indicate that chrysin inhibits MMP-9 activity in AGS cells via a reduction of the transcriptional efficiency.

### Role of AP-1 in PMA-induced MMP-9 expression

**Fig 2:**
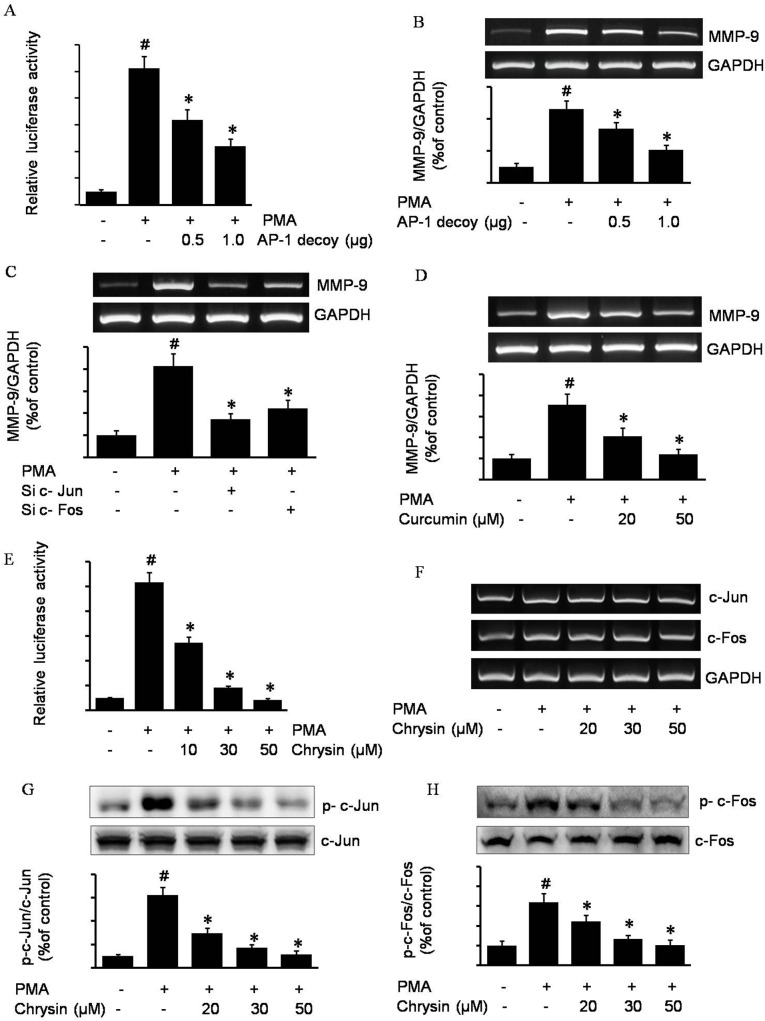
The role of AP-1 on MMP-9 expression and chrysin-inhibited MMP-9 expression by interfering with transcription factor AP-1 in AGS cells. The AP-1 decoy oligonucleotide was co-transfected with a pGL4-MMP-9 promoter into AGS cells. After incubation with 100 nM PMA for 4 h, MMP-9 promoter luciferase activity was examined using a luminometer (A), and MMP-9 mRNA was examined by RT-PCR (B). # *P*<0.05 versus control; * *P*<0.05 versus only PMA. C-Jun and c-Fos siRNA transfected AGS cells were incubated with 100 nM PMA for 4h, and after cell harvest MMP-9 mRNA was examined by RT-PCR. # *P*<0.05 versus control; * *P*<0.05 versus only PMA (C). AGS cells pretreated with the indicated concentration of curcumin for 1 h were incubated with 100 nM PMA for 4 h. After incubation, MMP-9 mRNA in the cell lysates was determined by RT-PCR. # *P*<0.05 versus control; * *P*<0.05 versus only PMA (D). The pGL3-AP-1 transfected cells were pretreated with the indicated concentrations of chrysin for 1 h, and then were incubated with 100 nM PMA for 4 h, and AP-1 luciferase activity was determined using a luminometer. # *P*<0.05 versus control; * *P*<0.05 versus only PMA (E). AGS cells pretreated with the indicated concentrations of chrysin for 1 h were incubated with 100 nM PMA for 4 h. After incubation, c-Jun and c-Fos mRNA in the cell lysates was determined by RT-PCR (F). Cells pretreated with the indicated concentrations of chrysin were incubated with 100 nM PMA, and cell lysates were analyzed for the phosphorylated c-Jun (G), phosphorylated c-Fos (H), total c-Jun and total c-Fos by western blot analysis. # *P*<0.05 versus control; * *P*<0.05 versus only PMA. The above data represents the mean ± SD from triplicate measurements.

As reported by previous literature, AP-1 is the most important transcription factor in MMP-9 expression [17]. In this study, to confirm that AP-1 is critically required in this course, we employed an AP-1 decoy phosphorothioated double-stranded oligodeoxynucleotide (ODNs) and pGL3-AP-1 luciferase plasmid to co-transfect into the AGS cells. The co-transfected cells were treated with PMA, and the luciferase activities were determined using a luminometer. As shown in Fig 2A, the PMA-induced MMP-9 promoter luciferase activities were inhibited by the AP-1 decoy. Consistently, AP-1 decoy also inhibited PMA-induced MMP-9 mRNA expression (Fig 2B). It was shown that knocking down the AP-1 component c-Fos and c-Jun also decreased PMA-induced MMP-9 expression (Fig 2C). Moreover, curcumin, an AP-1 inhibitor, also was employed to check the role of AP-1 in PMA-induced MMP-9 expression. As the RT-PCR data illustrates, curcumin inhibited PMA-induced MMP-9 expression in a dose-dependent manner (Fig 2D). The above data indicated that AP-1 is the essential factor in MMP-9 expression. After demonstrating the crucial role of AP-1 in the high levels of MMP-9 expression, we hypothesized that the mechanism of chrysin inhibition of MMP-9 expression was through the suppression of AP-1 activity. Subsequently, AGS cells transfected with AP-1 luciferase reporter plasmids were pretreated with chrysin, and then stimulated by PMA. As shown in Fig 2E, chrysin suppressed PMA-induced AP-1lucifease activity in a dose-dependent manner, which indicated that chrysin has an ability to decrease AP-1 activity.

### Chrysin inhibits PMA-induced MMP-9 expression by inhibiting transcription factor AP-1 via the suppression of c-Jun and c-Fos phosphorylation

It is well known that c-Jun and c-Fos are components of the AP-1 pathway[18]. We wanted to determine the mechanism behind chrysin’s inhibitory effect on AP-1, and investigated whether chrysin actually exerted its effect on c-Jun and c-Fos. We measured the amount of total mRNA of both c-Jun and c-Fos by RT-PCR. As shown in Fig 2F, chrysin did not inhibit the expression levels of c-Jun and c-Fos mRNA. Next, to test if chrysin inhibits the activation of c-Jun and c-Fos, western blotting was performed to monitor the phosphorylated c-Jun and c-Fos. Fig 2G and 2F shows that chrysin did suppress the PMA-induced c-Jun and c-Fos phosphorylation in a dose-dependent manner.

### Chrysin inhibits c-Jun and c-Fos phosphorylation by blocking JNK and ERK signaling pathway

**Fig 3:**
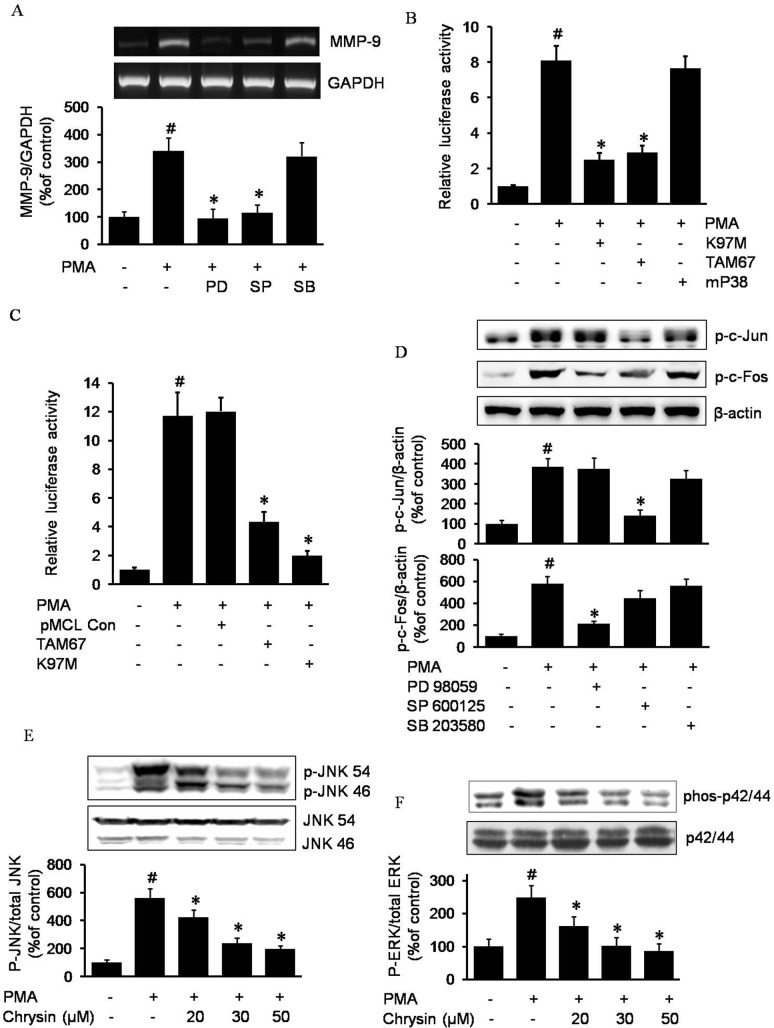
The role of MAPKs on MMP-9 expression and chrysin-inhibited MMP-9 expression by blocking JNK/c-Jun and ERK/c-Fos pathway in AGS cells. Cells pretreated with a concentration of 50μM PD98059 (PD), 50μM SP600125 (SP) or 20 μM SB203580 (SB) for 1 h were incubated with 100 nM PMA for 4 h. After incubation, MMP-9 mRNA in the cell lysates was determined by RT-PCR. # *P*<0.05 versus control; * *P*<0.05 versus only PMA (A). Expression vectors encoding a dominant negative mutant MEK (K97M), mutant JNK (TAM67), or mutant p38 MAPK (mP38) were co-transfected with pGL4-MMP-9 promoter constructs into AGS cells. After incubation with 100 nM PMA for 4 h, luciferase activity was determined using a luminometer. The data represents the mean ± SD from triplicate measurements. # *P*<0.05 versus control; * *P*<0.05 versus only PMA (B). Expression pMCL empty vector, encoding a dominant negative mutant MEK (K97M) or mutant JNK (TAM67) was co-transfected with pGL3-AP-1 luciferase construct into AGS cells. After incubation with 100 nM PMA for 4 h, luciferase activity was determined using a luminometer. The data represents the mean ± SD from triplicate measurements. # *P*<0.05 versus control; * *P*<0.05 versus only PMA (C). AGS cells pretreated with 50 μM PD98059, 50 μM SP600125, and 20 μM SB203580 were incubated with 100 nM PMA, and cell lysates were analyzed for the phosphorylated c-Jun and phosphorylated c-Fos by western blot analysis. # *P*<0.05 versus control; * *P*<0.05 versus only PMA (D). AGS cells pretreated with indicated concentration of chrysin were incubated with 100 nM PMA, and cell lysates were analyzed for the phosphorylated JNK1/2 and phosphorylated p42/44 by western blot analysis. # *P*<0.05 versus control; * *P*<0.05 versus only PMA (E and F).

We next investigated how chrysin suppressed c-Jun and c-Fos phosphorylation. Due to the important role MAPK plays in the activation of AP-1, we investigated the role of the MAPK pathway in MMP-9 expression. As shown in Fig 3A, among the MAPKs inhibitors, PD98059 (ERK1/2 inhibitor), SP600125 (JNK1/2 inhibitor), and SB203580 (p38 MAPK inhibitor), only PD98059 and SP600125 were able to inhibit PMA-induced MMP-9 expression, which indicated that the ERK1/2 and JNK1/2 pathways may be crucial for PMA-induced MMP-9 expression. Further, with the MEK-dominant negative plasmid pMCL-K97M, JNK-dominant negative plasmid pMCL-TAM67 and p38 MAPK-dominant negative plasmid pMCL-mP38, we confirmed the critical roles of ERK and JNK in MMP-9 expression in gastric cancer AGS cells (Fig 3B). Moreover, as shown in Fig 3C, ERK1/2 and JNK1/2 are critically required for AP-1 activation by PMA, demonstrated through our experiment with MEK and JNK dominant negative plasmids. We next checked the roles of JNK1/2 and ERK1/2 in c-Jun and c-Fos activation. As shown in Fig 3D, SP600125 and PD98059 suppressed PMA-induced c-Jun and c-Fos phosphorylation, respectively, indicating that JNK1/2 is critically upstream of c-Jun, while ERK1/2 is critically upstream of c-Fos. Since we discovered that JNK1/2 and ERK1/2 played essential roles in MMP-9 expression via AP-1, we hypothesized that chrysin may work through an inhibition of JNK1/2 or ERK1/2 to suppress MMP-9 expression. To verify our hypothesis, western blotting was performed to determine the effect of chrysin on JNK1/2 or ERK1/2 phosphorylation. As Fig 3E and 3F show, the PMA treatment led to a remarkable increase in JNK1/2 and ERK p42/44 phosphorylation; however, in the presence of chrysin, PMA-induced JNK1/2 and ERK1/2 phosphorylation was inhibited in a dose-dependent manner.

### Effect of chrysin on cell invasiveness

**Fig 4:**
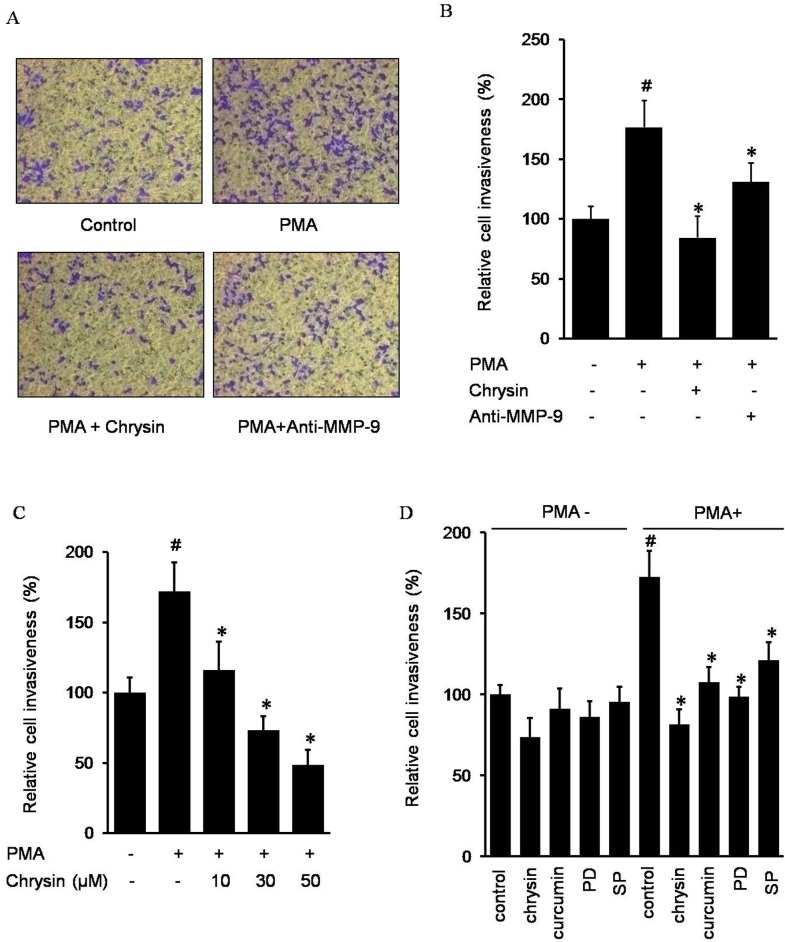
Chrysin inhibits AGS cells invasion by suppressing MMP-9. Cells were incubated with 50 nM PMA in the presence or absence of 30 μM chrysin or 200 ng/ml MMP-9 antibody in BIOCOAT Matrigel apparatus for 24 h (A and B). Cells were incubated with 50 nM PMA in the presence of 0–50 μM chrysin (C). Cells were incubated with 50 nM PMA in the presence of 30 μM chrysin, 20 μM curcumin, and either 30 μM PD98059 (PD) or 30 μM SP600125 (SP) (D). After incubation, the cells invaded the undersurface of the chambers and were counted using a phase contrast light microscope after staining with a Diff-Quick Stain Kit. The data represents the mean ± SD from triplicate measurements. # *P*<0.05 versus control; * *P*<0.05 versus only PMA.

It has been known that high levels of MMP-9 expression are important for the invasive phenotype of cancer cells. To examine the effect of chrysin on PMA-induced cell invasion, we examined cell invasion through a modified Boyden invasion chamber. AGS cells incubated in PMA resulted in an increased number of invaded cells which passed through the artificial matrigel. However, in the presence of chrysin or an MMP-9 antibody, the number of invaded cells decreased, indicating chrysin may suppress the PMA-induced cell invasiveness by inhibiting MMP-9 expression (Fig 4A and 4B). In addition, Fig 4C showed chrysin decreased PMA-induced AGS invasion in a dose-dependent manner. Next, we investigated the effect of the signaling inhibitor on PMA-induced AGS cell invasion. As shown in Fig 4D, chrysin, curcumin, PD98059 and SP600125 inhibited cell invasion induced by PMA, indicating that chrysin probably inhibits PMA-induced MMP-9 via AP-1 suppression, which results from blocking the JNK1/2 and ERK1/2 pathways.

## Discussion

Naturally occurring compounds with anti-cancer activities interfere with tumor development and the progression of cancer by inhibiting various mechanisms including cell migration, invasion, and metastasis. Chrysin, a natural flavonoid widely found in many plant extracts, honey, and propolis, has chemopreventive properties such as anti-proliferative and pro-apoptosis activities against various cancers [19,20]. However, the anti-invasion properties of chrysin have not been well studied in gastric cancer cells. It is well known that MMPs are the force behind the destruction of the extracellular matrix, as well as the main inducers of cell invasiveness. We investigated the inhibitory effects of chrysin on MMPs expression in gastric cancer cells. Chrysin inhibited MMP-9 expression (Fig 1) and other MMPs such as MMP-2 and MMP-7 (data not shown). The regulation mechanism by chrysin may be depended on each MMP, and in this study we focus on the MMP-9 regualtion.

PMA, phorbol-12-myristate 13-acetate, a protein kinase activator, is commonly used as a biomedical research tool in models of carcinogenesis. In this study, we discovered PMA increased MMP-9 activity through upregulation of its expression level. In the presence of chrysin, the PMA-induced MMP-9 decreased in a chrysin-dose-dependent manner (Fig 1A). However, it seems that chrysin inhibits MMP-9 not because of inhibition of MMP-9 enzyme activity, but due to its suppression of MMP-9 expression. To investigate the mechanism by which chrysin inhibits PMA-induced MMP-9 expression, we tried to discover the essential transcriptional factor(s) in the PMA- induced MMP-9 expression pathway. It has been reported that AP-1 is the positive regulator of MMPs: the AP-1 element located between -72 and -66 plays a major role in the transcriptional regulation of MMP-1 gene expression, and mutations of this element dramatically reduce the basal activity and responsiveness of the MMP-1 promoter to external stimuli [21]. In the MMP-9 promoter upstream regulation sequence, there are two AP-1 binding sites (-533 and -79) which play essential roles in MMP-9 expression in human Caski cells [22]. In our study, AP-1 decoy oligodeoxynucleotides, AP-1 inhibitor curcumin, c-Jun and c-Fos siRNA interfered with PMA-induced MMP-9 expression (Fig 2A–2D), which provided direct evidence that AP-1 is critically required to increase MMP-9 transcription in gastric cancer cells. Based on the above results, we inferred that chrysin inhibits MMP-9 expression by suppression of AP-1 activity. Fig 2E shows the verification of our speculation: chrysin can significantly decrease AP-1 activity in a dose-dependent manner. In line with our present discovery that chrysin inhibits AP-1 targeted gene MMP-9 via suppressing AP-1 activity, it also has been reported that chrysin inhibited other AP-1 target genes such as VEGF, COX-2 and ICAM-1 [23–25]. Consistent with other findings, it has been discovered that luteolin, a plant flavonoid with a similar structure to chrysin, significantly inhibits the LPS-induced DNA binding activity of AP-1 [26]. However, the inhibitory ability and mechanism of chrysin on AP-1 had not been fully investigated prior to this study. Therefore, we next studied the mechanism by which chrysin inhibits AP-1.

In addition to mediating MMP-9 expression, AP-1 also both induces the expression of invasion activators and represses invasion suppressors. As such, AP-1 is an essential transcriptional factor for the regulation of invasive-related genes [27]. AP-1 consists of either homodimers of Jun family members or heterodimers between members of the c-Fos and c-Jun families. Both c-Jun and c-Fos are phosphorylated and activated by the ERK, p38, and JNK kinase systems [28–30]. In order to investigate the mechanism of chrysin inhibition of AP-1, we investigated c-Jun and c-Fos, the two main components of AP-1. We discovered that chrysin inhibited c-Jun and c-Fos activation by blocking their respective phosphorylation. Consistent with our findings, some previous literature also reported that other flavones e.g. quercetin, decreased AP-1 activity through suppressing c-Jun’s translocation into the nucleus [31].

We were further interested in chrysin’s inhibitory mechanism on c-Jun and c-Fos. JNK (c-Jun N-terminal kinase), a member of the MAPK (mitogen-activated protein kinase) family that regulates a range of biological processes implicated in tumorigenesis, is considered to be the essential kinase of c-Jun activation [32]. ERK, extracellular signaling-regulated kinase, plays a role in the regulation of various cellular processes such as proliferation, differentiation, and development [33], and has been reported to be the important kinase regulating c-Fos activation [34]. In the present study, we find direct evidence in gastric cancer AGS cells that JNK is critically required for c-Jun activation, while ERK is the critical kinase for c-Fos activation (shown in Fig 3D).

Based on the above conclusion, in order to further investigate chrysin’s mechanism of suppressing MMP-9, we discovered that chrysin had an ability to block JNK1/2 and ERK1/2 phosphorylation (Fig 3E and 3F). In accordance with this finding, the inhibitory effects of other flavones on ERK1/2 also have been reported.

Liu et al. reported that apigenin, a polyphenolic flavone (4,5,7-trihydroxyflavone), found in a variety of herbs and green leafy vegetables, inhibits cell migration via the suppression of ERK1/2 phosphorylation in human bladder smooth muscle cells [35]. It has also been discovered by Ishikawa et al. that H_2_O_2_ increases apoptosis, leading to the rapid phosphorylation of JNK, ERKs, and p38 MAPK, while quercetin can abrogate the activation of these three MAPKs in response to H_2_O_2_ [36].

**Fig 5:**
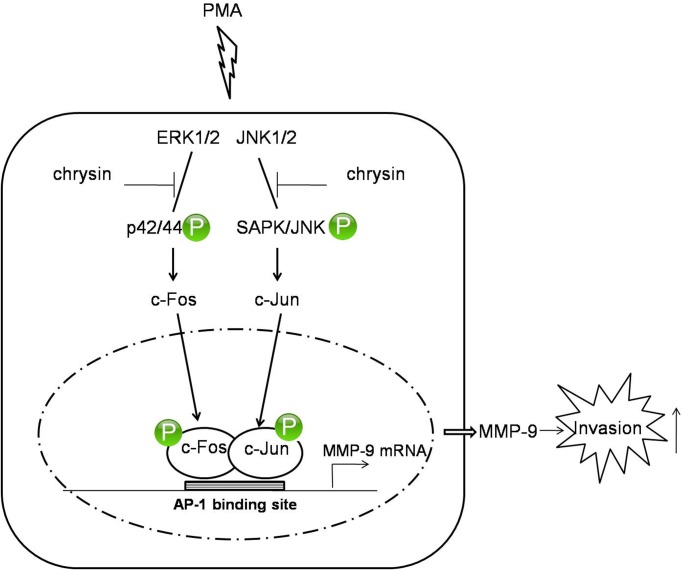
Schematic model of the signaling pathways involved in chrysin inhibiting PMA-induced MMP-9 expression. PMA stimulates ERK1/2 and JNK1/2 phosphorylation, in turn activating c-Fos and c-Jun, respectively. The phosphorylated c-Fos and c-Jun are translated into the nucleus, where they cooperatively bind to AP-1 binding site, thereby triggering MMP-9 gene expression. The higher level of MMP-9 expression enhances the degradation of the matrix and increases the cell invasiveness. In this model, chrysin acts as an inhibitor of the JNK1/2 and ERK1/2 signaling pathways, and decreases PMA-induced MMP-9 via suppressing AP-1 activation.

In the present study, we investigated the inhibitory effect of chrysin on MMP-9 expression and revealed the underlying mechanism in gastric cancer AGS cells for the first time. Based on our findings, we describe a hypothetical schematic model illustrating the mechanism of chrysin inhibiting MMP-9 expression, as shown in Fig 5. Further studies should be undertaken to investigate if there is an essential enzyme upstream of both JNK1/2 and ERK1/2, critically controlling JNK1/2 and ERK1/2 phosphorylation. If so, chrysin may directly interact with the essential enzyme, which should be thoroughly investigated as a potential anti-cancer therapeutic.

Various adjuvant therapeutic strategies to suppress tumor metastasis and prevent tumor recurrence have been explored and provide many options for patients’ recoveries. Recently, the use of natural phytochemicals has received extensive study for cancer prevention [37]. Moreover, dietary intake of phytochemicals has recently received attention in gastric cancer prevention [38]. In this study, we found that chrysin-treated cells can decrease cancer invasiveness via inhibiting MMP-9 expression through the suppression of the JNK/c-Jun and ERK/c-Fos signaling pathways. These findings may provide useful evidence for developing future anti-cancer therapeutics for gastric cancer.
